# Predictors for Fear of Cancer Recurrence in Breast Cancer Patients Referred to Radiation Therapy During the COVID-19 Pandemic: A Multi-Center Cross-Section Survey

**DOI:** 10.3389/fonc.2021.650766

**Published:** 2021-07-26

**Authors:** Jinrong Xie, Weixiang Qi, Lu Cao, Yuting Tan, Jin Huang, Xiaodong Gu, Bingguang Chen, Peipei Shen, Yutian Zhao, Ying Zhang, Qingwen Zhao, Hecheng Huang, Yubin Wang, Haicheng Fang, Zhenjun Jin, Hui Li, Xuehong Zhao, Xiaofang Qian, Feifei Xu, Dan Ou, Shubei Wang, Cheng Xu, Min Li, Zefei Jiang, Yu Wang, Xiaobo Huang, Jiayi Chen

**Affiliations:** ^1^ Department of Radiation Oncology, Ruijin Hospital Affiliated to Shanghai Jiaotong University School of Medicine, Shanghai, China; ^2^ Guangdong Provincial Key Laboratory of Malignant Tumor Epigenetics and Gene Regulation, Medical Research Center, Sun Yat-sen Memorial Hospital, Sun Yat-sen University, Guangzhou, China; ^3^ Department of Radiation Oncology, The Third Affiliated Hospital of Soochow University, Changzhou, China; ^4^ Department of Radiation Oncology, Shanxi Provincial Cancer Hospital, Taiyuan, China; ^5^ Department of Oncology, Yunfu People’s Hospital, Yunfu, China; ^6^ Department of Radiation Oncology, Affiliated Hospital of Jiangnan University, Wuxi, China; ^7^ Oncology Center, Affiliated Hospital of Guangdong Medical College, Zhanjiang, China; ^8^ Department of Oncology, Datong Second People’s Hospital, Datong, China; ^9^ Department of Radiation Oncology, Shantou Central Hospital, Shantou, China; ^10^ Department of Radiation Oncology, The Third Affiliated Hospital of Wenzhou Medical University, Wenzhou, China; ^11^ Radiation Oncology Center, Puning Overseas Chinese Hospital, Jieyang, China; ^12^ Department of Radiation Oncology, Changshu No.1 People’s Hospital Affiliated to Soochow University, Changshu, China; ^13^ Oncology Center, Yuncheng First Hospital, Yuncheng, China; ^14^ Department of Oncology, Lin-fen Central Hospital, Linfen, China; ^15^ Department of Breast Oncology, The Fifth Medical Center of PLA General Hospital, Beijing, China; ^16^ Chinese Society of Clinical Oncology (CSCO), Beijing, China; ^17^ Department of Breast Tumor Radiotherapy, Sun Yat-sen Memorial Hospital, Sun Yat-sen University, Guangzhou, China

**Keywords:** breast cancer, COVID-19, cross-section study, delayed radiotherapy initiation, fear of cancer recurrence (FCR), quality of life, radiotherapy interruption

## Abstract

**Objective:**

The outbreak of COVID-19 pandemic has greatly impacted on radiotherapy (RT) strategy for breast cancer patients, which might lead to increased distressing psychological symptoms. We performed a multi-center cross-section survey to investigate prevalence of fear of cancer recurrence (FCR) and predictors for FCR in patients referred to RT during pandemic.

**Methods:**

542 patients were consecutively enrolled from three regions in China including Yangtze Delta River Region, Guangdong and Shanxi province. Patients’ characteristics were collected using an information sheet, Fear of progression questionnaire-short form, Hospital Anxiety/Depression Scale and EORTC QLQ-C30. The hierarchical multiple regression models were performed.

**Results:**

488 patients with complete data were eligible. The RT strategy was affected in 265 (54.3%) patients, including 143 with delayed RT initiation, 66 believing to have delayed RT initiation but actually not, 24 with RT interruptions, 19 shifting to local hospitals for RT and the remaining 13 influenced on both RT schedule and hospital level. The model explained 59.7% of observed variances in FCR (p<0.001) and showed that influence of RT strategy had significantly impacted on FCR (△R2 = 0.01, △F=2.966, p=0.019). Hospitals in Shanxi province (β=-0.117, p=0.001), emotional function (β=-0.19, p<0.001), social function (β=-0.111, p=0.006), anxiety (β=0.434, p<0.001) and RT interruption (β=0.071, p=0.035) were independent predictors.

**Conclusions:**

RT strategy for breast cancer patients was greatly influenced during pandemic. RT interruption is an independent predictor for high FCR. Our findings emphasize the necessity to ensure continuum of RT, and efforts should be taken to alleviate FCR through psychological interventions.

## Introduction

From late December in 2019, severe outbreak of the novel coronavirus disease (COVID-19) pandemic in China has greatly impacted the routine medical practice for cancer patients, which has brought great psychological pressure on patients, especially for those who undergo anti-cancer treatments. Cancer patients are generally at systemic immunosuppressive status (whether caused by the disease or anti-cancer therapies), therefore are more susceptible to COVID-19 infection and more likely to have morbidity than the general population (1–3). Several guidelines on recommendations for the management of cancer patients during pandemic have suggested the importance of maintaining normal mental health (4–6). However, recently published studies mostly focused on psychological symptoms caused by COVID-19 among the medical staffs and general population (7–9), limited data regarding psychological status of cancer patients during pandemic is available, especially for patients with affected anti-cancer treatment due to COVID-19.

Breast cancer (BC) is the most commonly diagnosed cancer in women worldwide (10). Approximately 27% of BC patients in China receive radiotherapy (RT) as part of primary treatment based on retrospective epidemiological study (11). Different from surgery and chemotherapy, patients who received RT need to attend daily with the standard regimen of 4-6 weeks. Overall RT time has been found to impact on local control and survival, which emphasizes the necessity to ensure the continuum of RT (12). However, the timely delivery of RT during pandemic has unique challenges. During the peak of pandemic, a series of quarantine measures were taken to control the spread of COVID-19, especially in high-risk areas (13, 14). Screenings including chest computed tomography (CT) scan were required repeatedly for hospitalization in some hospitals (13–15). These measures would limit access to scheduled anti-cancer treatments and interfere with the RT strategy for patients. Under such situations, unplanned delay and/or interruption of RT were common, which would be associated with psychological stress of patients. Fear has been reported to be the dominant emotion among BC patients during pandemic (16). The fear can be categorized into two aspects: the general fear for COVID-19 and fear of cancer recurrence (FCR) due to delay or impairment of standard protocol.

Currently, the COVID-19 continues to spread globally (17). A recent national survey in China reported that the significant delays in treatment for early BC patients were seen when compared with treatment before (18). However, data for detailed impact on RT strategy was not enough. Sponsored by Chinese Society of Clinical Oncology Breast Cancer (CSCO BC), this survey was conducted in three regions outside the center of pandemic (Hubei province) in China and aimed to understand the impact of alteration of RT strategy on psychological health in BC patients during outbreak of COVID-19.

## Methods

### Study Design and Participants

In this multi-center cross-section survey, eligible BC patients from 14 hospitals in China were enrolled to complete questionnaires. Patients were eligible if they had a confirmed pathological diagnosis of BC referred to RT during the COVID-19 pandemic (from 24th Jan to 30th Apr, 2020). The 14 hospitals were located in three representative regions of mainland China, with 5 hospitals in the Yangtze River Delta Region (eastern China), 5 in the Guangdong province (southern China, severely affected by COVID-19 behind to Hubei) and 4 in the Shanxi province (central China).

### Instruments

The questionnaires (see in the **Additional File 1**) included four parts as below.

#### Demographic Characteristics and Treatment Information

This part was showed in **Table 1**. Stage of tumor was classified as operable or recurrent (local-regional)/metastatic BC. According to the number of BC patients admitted for RT in one hospital during the past three years, “hospital volume” was divided into three categories: <100, 100-499, ≥500 BC patients per year.

**Table 1:** Characteristics of demographic, treatment and influence of COVID-19(N=488).

Values		Total (*N*=488) *N* (%)	The Yangtze River Delta region (*N*=191) *N* (%)	The Guangdong province (*N*=193) *N* (%)	The Shanxi Province (*N*=104) *N* (%)	*p*
Age, y						0.001*
	≤40	88 (18.0)	40 (20.9)	33 (17.1)	15 (14.4)	
	41~60	313 (64.1)	103 (53.9)	131 (67.9)	79 (76)	
	>60	87 (17.8)	48 (25.1)	29 (15)	10 (9.6)	
Sex						0.521
	Female	486 (99.6)	190 (99.5)	193 (100)	103 (99)	
	Male	2 (0.4)	1 (0.5)	0 (0)	1 (1)	
Employment status						0.071
	Employed	168 (34.4)	75 (39.3)	66 (34.2)	27 (26)	
	unemployed	320 (65.6)	116 (60.7)	127 (65.8)	77 (74)	
Education						0.114
	High school and below	341 (69.9)	125 (65.4)	140 (72.5)	76 (73.1)	
	bachelor	138 (28.3)	59 (30.9)	52 (26.9)	27 (26)	
	Master and above	9 (1.8)	7 (3.7)	1 (0.5)	1 (1)	
Marriage						0.098
	Single	13 (2.7)	8 (4.2)	5 (2.6)	0 (0)	
	Married	474 (97.1)	183 (95.8)	187 (96.9)	104 (100)	
	widowed	1 (0.2)	0 (0)	1 (0.5)	0 (0)	
Hospital volume						<0.001*
	<100 BC cases	54 (11.1)	0 (0)	42 (21.8)	12 (11.5)	
	100~499 BC cases	165 (33.8)	102 (53.4)	40 (20.7)	23 (22.1)	
	≥500 BC cases	269 (55.1)	89 (46.6)	111 (57.5)	69 (66.3)	
Stage of tumor						<0.001*
	operable BC	446 (91.4)	186 (97.4)	174 (90.2)	86 (82.7)	
	recurrent or metastatic BC	42 (8.6)	5 (2.6)	19 (9.8)	18 (17.3)	
Types of recurrent or metastatic BC						0.487
	Local	27 (64.3)	2 (40)	14 (73.7)	11 (61.1)	
	distant	10 (23.8)	2 (40)	4 (21.1)	4 (22.2)	
	Local + distant	5 (11.9)	1 (20)	1 (5.3)	3 (16.7)	
Surgery in early BC						0.004*
	Mastectomy	220 (49.3)	82 (44.1)	82 (47.1)	56 (65.1)	
	BCS	226 (50.7)	104 (55.9)	92 (52.9)	30 (34.9)	
Chemotherapy						0.444
	Yes	414 (84.8)	162 (84.8)	160 (82.9)	92 (88.5)	
	No	74 (15.2)	29 (15.2)	33 (17.1)	12 (11.5)	
Target therapy						0.11
	Yes	128 (26.2)	53 (27.7)	56 (29)	19 (18.3)	
	No	360 (73.8)	138 (72.3)	137 (71)	85 (81.7)	
Endocrine therapy						<0.001*
	Yes	329 (67.4)	130 (68.1)	146 (75.6)	53 (51)	
	No	159 (32.6)	61 (31.9)	47 (24.4)	51 (49)	
RT procedure						<0.001*
	Completed	143 (29.3)	76 (39.8)	50 (25.9)	17 (16.3)	
	Undergoing	268 (54.9)	77 (40.3)	133 (68.9)	58 (55.8)	
	Planned to RT	77 (15.8)	38 (19.9)	10 (5.2)	29 (27.9)	
Influence of RT schedule						<0.001*
	Special normal	73 (15)	16 (8.4)	26 (13.5)	31 (29.8)	
	Interruption	24 (4.9)	0 (0)	21 (10.9)	3 (2.9)	
	Delay	149 (30.5)	20 (10.5)	95 (49.2)	34 (32.7)	
	normal	242 (49.6)	155 (81.2)	51 (26.4)	36 (34.6)	
Change of hospital level						0.365
	Down	32 (6.6)	11 (5.8)	11 (5.7)	10 (9.6)	
	Up or no change	456 (93.4)	180 (94.2)	182 (94.3)	94 (90.4)	

*p < 0.05.

BC, breast cancer; BCS, breast conserving surgery; RT, radiotherapy.

#### Influence of COVID-19 Pandemic on RT

A study specific set of questions were formulated to collect information about the influence of pandemic on RT:

1) What stage of RT procedure were you on during the Spring Festival of 2020 (peak of pandemic in China) and today, respectively? (1) planned to RT (2) undergoing (3) completed;
2) Was your RT schedule modified during the pandemic? (1) continued as planned (2) interrupted (3) others;
3) How many days was your RT interrupted?
4) Did you think that your RT schedule had been impacted by pandemic? Which of the following reasons account for the interruption or delayed RT: (1) aggressive quarantine measures (2) restricted number of cancer patients admitted for hospital under the constraints of social distancing and local guidelines (3) personal reason, afraid of being infected by COVID-19 (4) declined to receive repeated COVID-19 screenings including chest CT scan (5) restriction of number of beds in hospital due to COVID-19 (6) others;
5) If your RT schedule was impacted by COVID-19, could you tell the detailed time?
6) Whether you and/or your family members were diagnosed as COVID-19?
All questions were single-choice except reasons for the influences by pandemic.

#### Fear of Progression Questionnaire⁃Short Form (FoP-Q-SF)

The FoP-Q was a validated and reliable instrument to measure FCR in cancer patients (19). It was a short-item version with 12 items scored on a 5-point Likert Scale ranging from never (1) to very often (5) (20). The sum score ranges from 12 to 60 with higher score indicating higher FCR. The high-level FCR was defined as sum score ≥34, which was recommended for psychological interventions. The version of the questionnaire had been verified in Chinese population with a high internal consistency (Cronbach’s alpha = 0.88) (21).

#### Hospital Anxiety and Depression Scale (HADS) and EORTC QLQ-C30

The two questionnaires were used to assess the status of anxiety, depression and quality of life (QoL) during pandemic. The HADS is one of most frequently used scales to assess anxiety and depression, which consists of two seven-item subscales to assess anxiety and depression with higher scores meaning higher levels (22). EORTC QLQ-C30 contains five functional scales (physical, role, emotional, cognitive and social function) and global QoL (23). All scales had been converted into standard scores ranging from 0 to 100, with higher scores indicating better function and QoL. Both Chinese version HADS and EORTC QLQ-C30 had been validated with a high internal consistency (24, 25).

### Procedure and Data Collection

Between 9^th^ April and 30^th^ April 2020, a consecutive sampling method was used to enrolled participants. Eligible patients could choose to complete the questionnaires either online through Wenjuanxing (www.wjx.cn) or on paper. Informed consents were provided electronically or on paper prior to registration. The present study was approved by the Ethical Committee of Ruijin Hospital, Shanghai Jiao Tong University, school of medicine.

### Outcomes

In the study, we hypothesized that timely delivery of RT would be impacted during pandemic, which might affect FCR. We also assessed the prevalence of FCR and investigated other predictors for FCR in patients referred for RT. Additionally, we investigated associations between FCR and anxiety/depression or QoL. The influence of pandemic on RT schedule was divided into four categories: normal, delay, interruption and “special normal”. According to protocols of EORTC 22922 (26) and ABCSG-53 (27), “delay” was defined as >12 weeks from surgery to RT in patients without chemotherapy or >8 weeks from the last dose of chemotherapy to RT in patients with adjuvant chemotherapy or >8 weeks from surgery to RT in patients receiving neoadjuvant chemotherapy (NAC). “Special normal” was defined as those patients who believed to have delayed RT initiation caused by COVID-19 but their RT delivery was actually not delayed according to the aforementioned definition. “Interruption” was defined as any unplanned treatment breaks. All other RT schedules were classified into “normal”.

Another type of influence on RT strategy subsequent to COVID-19 pandemic was that patients shifted planned RT hospital from Grade-A tertiary hospitals to local hospitals, which was defined as “down” in our analysis, while other situations of hospitals shifting were classified as “up or no change”.

### Statistical Analysis

Descriptive analysis was performed to characterize the demographic characteristics, treatment, influences of RT strategy during pandemic. χ² test was used to compare the differences between three regions. Univariable analyses of FCR were performed in a one-way analysis of variance (ANOVA) or student t-test. The pairwise comparison was calculated with Least Significant Difference test. If inhomogeneity of variances assumption was detected, Welch’s and Dunnett’s T3 test would be performed. Pearson correlation analyses were conducted to explore correlations of FCR with HADS or QLQ-C30, respectively. Subsequently, hierarchical regression model was performed to identify predictors for FCR. Candidate variables with *P*<0.2 in ANOVA, t-test or Pearson correlation analyses were included and defined as explanatory variables in the model.

The hierarchical regression model was divided into four blocks:

Block 1: Treatment-related information (hospital regions, hospital volume of RT, stage of tumor and endocrine therapy); In the “hospital regions” variable, Guangdong province was performed as references because it was the most serious region with COVID-19 infection among three regions.
Block 2: Functional scales and global QoL in QLQ-C30;
Block 3: Anxiety and depression scores according to HADS questionnaire;
Block 4: Influence of RT strategy during pandemic (influence of RT schedule and changes of hospital levels);

The variables inflation factor <5 indicated that there was no serious multicollinearity.

To explore the correlations between the time to RT from the last dose of CT and FCR, sub-group analysis for operable BC patients with adjuvant chemotherapy was performed with Pearson correlation analyses. A two-side *p*-value <0.05 was considered to be statistically significant and all analysis was performed by SPSS 21.0 software (IBM corporation, USA).

## Results

A total of 641 eligible BC patients were recruited in this multi-center cross section survey while 542 BC patients completed the questionnaires. Of them, 54 questionnaires were excluded due to different reasons showed in **Figure S1** (see in **Additional File 2**). Finally, 488 complete validated questionnaires were included and the response rate was 76.1%.

### Demographic and Medical Characteristics

**Figure 1:**
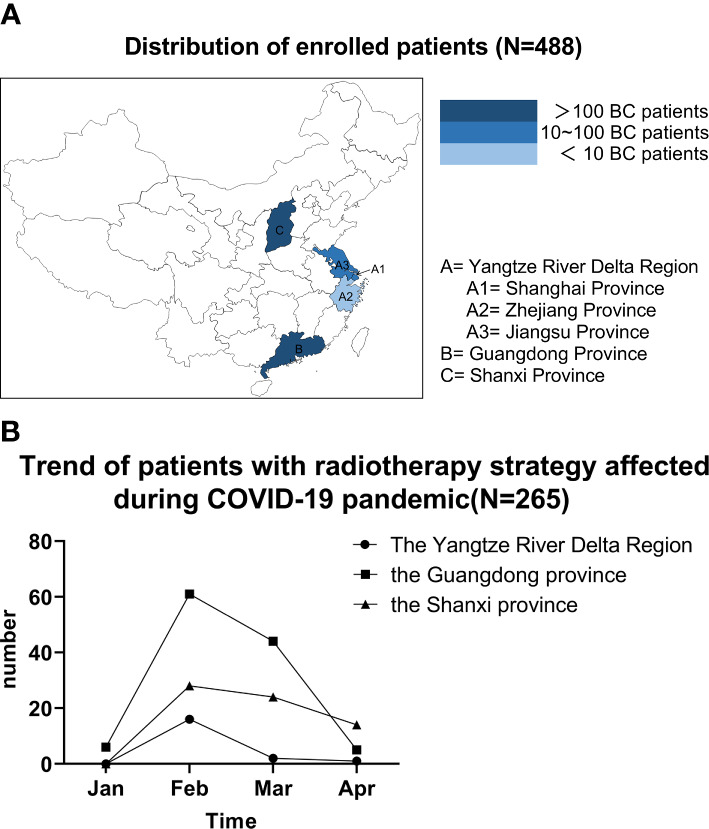
**(A)** Distribution of enrolled patients (N=488). **(B)** Trend of patients with radiotherapy strategy affected during COVID-19 pandemic (N=265); **(A)** Number of patients in the Yangtze River Delta Region, Guangdong, Shanxi were 191, 193 and 104, respectively. For the Yangtze River Delta Region, most patients were from Shanghai and Jiangsu Province while only 9 patients came from Zhejiang where number of newly confirmed COVID-19 patients was higher than Shanghai and Jiangsu Province. **(B)** RT strategy were affected in late January and February in most of patients, which was peak of COVID-19 pandemic in China. BC, breast cancer.

The median(range) age was 50 (27-87) years. Number of patients in the Yangtze River Delta Region, Guangdong, Shanxi were 191, 193 and 104, respectively. For the Yangtze River Delta Region, 89 (46.6%) and 93 (48.7%) patients were from Shanghai and Jiangsu Province respectively, only 9 (4.7%) patients came from Zhejiang where the number of newly confirmed COVID-19 patients was high (**Figure 1A**).

Demographic and clinical characteristics were well balanced among three regions except age, hospital volume, tumor stage, surgery and endocrine therapy (**Table 1**). Moreover, none of patients and their family members had been diagnosed as COVID-19.

### Influence on RT Strategy During Pandemic

The RT strategy was affected in 265 (54.3%) patients during pandemic, including 143 with delayed RT initiation, 66 believing to have delayed RT initiation but actually not (special normal), 24 (4.9%) with RT interruptions, 19 shifting to local hospitals for RT, and the remaining 13 patients influenced on both RT schedule and hospital level (**Figure S1** see in **Additional File 2**). The median(range) time of delayed RT initiation was 34 days (1-117 days). For 24 patients with interrupted RT, median (range) fractions prior to interruption and length of RT interruptions were 10 (4-27) fractions and 20 (10-33) days.

The influences of RT schedule by pandemic in Guangdong were more serious than other two regions (*p*<0.001, **Table 1**). RT strategy were affected in late January and February in most of patients, which was peak of COVID-19 pandemic in China (**Figure 1B**). As shown in **Table S1** (see in **Additional File 2**), the most common reason for RT strategy modification was restricted number of cancer patients admitted for hospital under the constraints of social distancing and local guidelines (62.3%), followed by restriction of number of beds in hospitals due to COVID-19 (34.7%). And 4.9% of these 265 patients had their RT schedule affected because of declining to repeated COVID-19 screenings.

### Influence on the Time to RT

Three hospitals with highest number of participants in each region further provided data of the time to RT after last dose of adjuvant chemotherapy, which serves as representative of the corresponding region. The mean time to RT in the representative hospital of Guangdong and Shanxi province were 74.7 and 61.9 days, which were 32 days and 22.2 days delay in comparisons with the time to RT in 2019 (*p＜0.001* and *p=0.006*, respectively) (**Table S2**, see in the **Additional File 2**). There was no significant difference in distribution of the time to RT in the Yangtze River Delta Region.

### Fear of Cancer Recurrence

The mean score of FCR was 24.83 [standard distance (SD)=8.554], and 84 (17.2%) patients experienced high-level FCR. The single-item analysis showed that the two most common fears were item 10: worrying about medications could damage the body (Mean=2.7, SD=1.114) and item 11: worrying about what will become of the family (Mean=2.44, SD=1.134). Excepting for item 6: being afraid of the possibility that the children could contract cancer, the mean FCR scores of other 11 items among patients with interrupted RT were higher than other three groups (**Figure S2** see in **Additional File 2**).

### Univariable Analysis of FCR

In univariable analyses (**Table 2** and **Figure S3** see in **Additional File 2**), FCR were higher in patients who received RT in hospitals with less than <100 BC cases per year (*p*<0.05). We also found that mean scores of FCR for patients in Guangdong were significantly higher than in Shanxi (26.0 ± 8.742 vs 23.57 ± 9.5, *p*=0.019). No significant difference was found between operable and recurrent/metastatic BC patients, although 38.1% of recurrent/metastatic BC patients in our cohort had high-level FCR (*p*=0.154). In terms of influence on RT schedule during pandemic, a significant difference in FCR was observed among four groups (*p*<0.001). Out of 24 patients experienced RT interruption, 50% had high-level FCR. And 31.5% of patients with “special normal” showed high-level FCR, which was higher than those in normal (13.2%) or delayed (11.4%) groups. Moreover, FCR were higher in patients with RT hospital level “down” than those in the “up or unchanged” group(*p*=0.009). As for other variables, no significant differences in FCR were observed **(** **Table S3** see in **Additional File 2)**.

**Table 2:** Univariate analysis on associated factors with fear of cancer recurrence scores.

Factors		Whole cohort (*N*=488)				Patients with High-level FCR (*N*=84)	
		*N*	Mean	SD	*p*	*N*	%^a^
Hospital volume					0.006*		
<100 BC cases		54	29.04	10.233		16	29.6
100~499 BC cases		165	24.52	7.829		27	16.4
≥500 cases BC		269	24.18	8.409		41	15.2
Region					0.038*		
The Yangtze River Delta Region		191	24.34	7.676		29	15.2
Guangdong province		193	26	8.742		39	20.2
Shanxi province		104	23.57	9.5		16	15.4
Stage of tumor					0.154		
operable BC		446	24.62	8.328		68	15.2
recurrent or metastatic BC		42	27.05	10.532		16	38.1
Endocrine therapy					0.103		
Yes		329	25.27	8.856		60	18.2
No		159	23.92	7.842		24	15.1
Influence of RT schedule					<0.001*		
normal		242	23.96	7.854		32	13.2
Delay of RT		149	23.81	7.853		17	11.4
Interruption of RT		24	30.75	8.759		12	50
Special normal		73	27.88	10.555		23	31.5
Change of hospital level					0.009*		
Down		32	28.63	9.387		8	25
Up or no change		456	24.57	8.44		76	16.7

*p < 0.05.

^a^The percent, number of patients with high-level FCR/ total number of patients in each subgroup.

FCR, fear of cancer recurrence; BC, breast cancer; BCS, breast conserving surgery; RT, radiotherapy.

### Correlation Between FCR and Anxiety, Depression, QoL

There were significant negative correlations between FCR and all five functional scales (physical, role, emotional, cognitive and social) and global QoL in QLQ-C30 (r=-0.341~-0.598, p<0.001). In addition, anxiety and depression had positive correlations with FCR (r=0.577~0.701, p<0.001, **Table S4** see in **Additional File 2**).

### Hierarchical Multiple Regression

**Figure 2:**
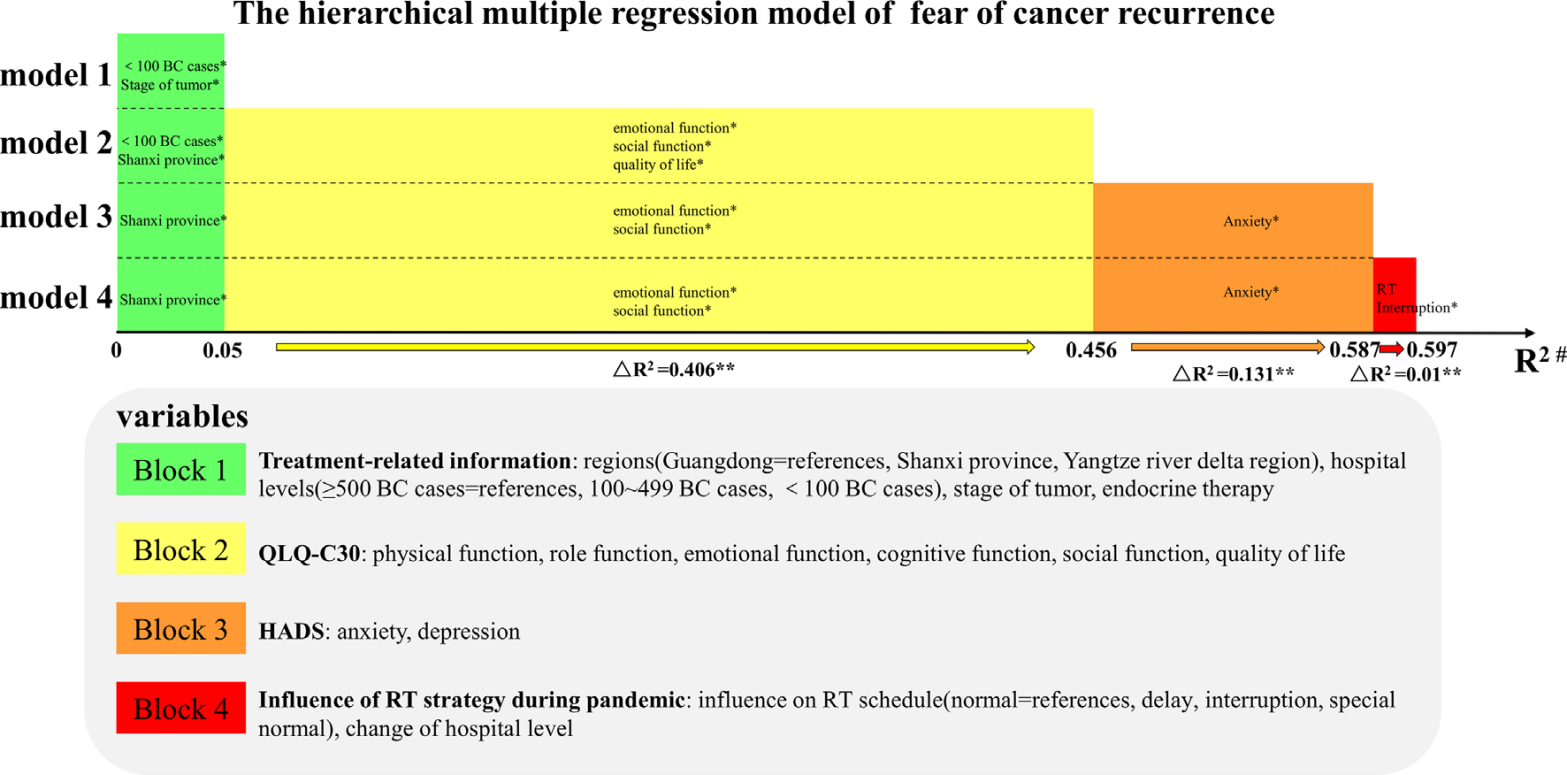
The hierarchical multiple regression model of fear of cancer recurrence. Candidate variables with P < 0.2 in ANOVA, t-test or Pearson correlation analyses were defined as explanatory variables and divided into four Block in the model: treatment-related information, functional scales and global QoL in QLQ-C30, anxiety and depression score according to HADS questionnaire, influence of RT strategy during pandemic. New variables in the corresponding Block will be added to the model in each step to explore the effect of adding variables to FCR. In Block 4, influence of RT schedule and change of hospital levels were included. The final model showed that an additional 1% of the variances in FCR was observed and changes of the model remained significant (△R2 = 0.01, △F=2.966, p=0.019). Finally, hierarchical multiple regression model explained 59.7% of the observed variances in FCR and influence of RT strategy during pandemic had significantly impact on FCR (△R2 = 0.01, △F=2.966, p=0.019). *variables of p < 0.05 in models; **p < 0.05 for changes of models; ^#^coefficient of determination for corresponding step. BC, breast cancer; RT, radiotherapy.

The results of hierarchical multiple regression were showed in **Figure 2** and **Table S5** (see in **Additional File 2**) Based on the results of univariable analyses, 4 treatment-related variables: hospital volume, regions, stage and endocrine therapy were introduced into Block 1. The result showed that these four treatment-related variables explained 5% of variances of FCR (△F=4.182, *p*<0.001). Only hospitals volume with <100 cases and recurrent/metastatic BC had a significant association with FCR (*p*<0.05) in model 1. In Block 2 and 3, we included all 5 functional scales, global QoL in QLQ-C30 and psychological factors (anxiety and depression). The increasing explained variances by 40.6% and 13.1% for high-level FCR were observed in model 2 (△F=59.055, *p*<0.001) and model 3 (△F=74.987, *p*<0.001), respectively.

In block 4, influence of RT schedule and change of hospital levels were included. The final model showed that an additional 1% of the variances in FCR were observed. Although the increased R^2^ for FCR was small, changes of the model remained significant (△R^2^=0.01, △F=2.966, *p*=0.019). Finally, hierarchical multiple regression model explained 59.7% of the observed variances in FCR and influence of RT strategy during pandemic had a significant impact on FCR (△R^2^=0.01, △F=2.966, *p*=0.019). In total, the final model indicated that hospitals located in Shanxi province (β=-0.117, *p*=0.001), emotional function (β=-0.19, *p<*0.001), social function (β=-0.111, *p*=0.006), anxiety (β=0.434, *p*<0.001) and interruptions of RT (β=0.071, *p*=0.035) were independent predictors for FCR.

## Discussion

In this multi-center cross-section survey, we found that RT strategy for breast cancer patients was greatly influenced during pandemic and RT interruption is an independent predictor for high FCR.

The outbreak of COVID-19 pandemic has significantly disrupted the routine medical treatment for BC patients worldwide, including the decline in screening mammography and surgery, delay in diagnosis and treatment, suspended clinical trials and so on (28–32). Similarly, our survey found that RT delivery has been influenced in more than half of enrolled BC patients (54.3%) during pandemic. Previous studies had reported that adverse effects on both local control and survival could be observed in BC if RT was prolonged for more than seven days in a five-week course of treatment due to interruptions (12, 33). It should be noted that a total of 24 patients experienced RT interruptions in our survey, although none of the 14 hospitals located in Hubei province where the center of pandemic was. In our study, the median length of RT interruptions was 20 days. Thus, compensatory measures should be taken to minimize the effects of prolongation of overall treatment time. The COVID-19 Pandemic Breast Cancer Consortium recommended additional boost in original treatment plan (5). These recommendations have been adopted for these interrupted RT patients in our survey by adjusting boost doses according to different fractionation regimens and gaps of interruptions. In our study, 30.5% BC patients encountered delayed RT which was defined in our study and 13.8% initiated RT over 12 weeks after last dose of chemotherapy. Trials have demonstrated that a delay up to 20 weeks from surgery did not impact the prognosis for patients with early stage, node negative, hormonal receptor positive with no chemotherapy (34, 35). However, evidences for acceptable interval from end of adjuvant chemotherapy to RT were limited (35, 36). A recent study of 989 operable BC patients reported that the time to RT after chemotherapy over 12 weeks negatively impact 5-year distant recurrence free survival and breast cancer specific survival (37). Long-term follow-up is needed to assess the prognosis of patients who experienced RT interruption or delay. In addition, guidelines on the management of treatment for BC patients during pandemic have been recommended by several authoritative cancer organizations (5, 36, 38, 39), which has provided selection criteria and prioritization of treatments for oncologists to make decisions during pandemic. As to the recommendations for RT, short course regimens are preferred in clinical practices, which will not only be associated with increased efficiency but also decreased events of interruptions and hospital visits.

In our study, 50% of patients with RT interruptions had high-level FCR, which indicates that unplanned RT interruptions significantly impact patients’ psychological emotions. The hierarchical multiple regression model further confirms that the interruption of RT (*p*=0.035) is an independent predictor for FCR, but not delayed RT initiation. One possible explanation is that most patients who experienced delayed RT initiation were not aware of the delay. In fact, our survey defines a subgroup as “special normal”, referring to those patients who believed to have delayed RT plans but actually not. In this sub-group, 31.5% patients show high-level FCR, much higher than that in the normal (13.2%) or delay (11.4%) group. These results imply that awareness of “delay” is a much more important predictor for FCR than the existence of delay, indicating that professional telemedicine is necessary to answer queries concerned by patients when the access to face-to-face clinic visits was limited during pandemic. Meanwhile, an interesting comparison of the prevalence of the “special normal” and actual “delay” group showed that the highest “delay” occurred in Guangdong province (49.2%), while the highest rate of “special normal” was found in Shanxi province (29.8%). The possible answer to this finding is that, since April 2019, the leading hospital in Guangdong province started to participate in our phase III trial of hypo-fractionated radiotherapy of node positive patients receiving regional nodes irradiation (the HARVEST study, NCT03829553). There is definition in accepted interval of RT to the end of adjuvant chemotherapy in the trial. It is very possible that the participation of the prospective trial helped standardization of clinical practice in an area which no strict consensus exists. This result implies the importance of patient narration based on scientific study, which brings professional recommendation that is more convincing in case of public health emergency.

Prior to our study, multiple studies have indicated that QoL is an important predictor for high-level FCR (40, 41). In consistent with these results, our study also found that all 5 functional scales (physical, role, emotional, cognitive and social) were negatively correlated with FCR. Emotional function (r=-0.103, *p<*0.001) and social function (r=-0.052, *p*=0.006) were independent predictors for high FCR. Additionally, patients in Shanxi were significantly associated with lower FCR compared with Guangdong, which was consistent with the level of pandemic. These results imply that extra attention should be paid to psychological status of BC patients referred for RT in regions with higher level of COVID-19 pandemic.

Until now, newly COVID-19 confirmed cases continue to be increasing worldwide. Strict quarantine measures are still necessary in order to control the spread of the pandemic. In this situation, a majority of BC patients referred for RT worldwide might face the same dilemma as in China during the peak of pandemic. Based on our findings, we recommend that psychological interventions should be applied to BC patients who experienced delayed RT initiation or RT interruptions, especially to those who feel anxious about delayed RT. Although traditional face-to-face social psychological interventions have been proved to be good tools to reduce FCR (42, 43), strict quarantine during pandemic are changing the way psychologists work. Imai F et al. (44) developed a newly smartphone problem solving therapy (PST) application to reduce FCR among BC survivors. The preliminary results showed that smartphone PST could significantly reduce FCR at 8 weeks when compared with baseline. More recently, West China Hospital has also developed a new psychological crisis intervention model by using internet technology to deal with psychological problems involved in the COVID-19 pandemic (45). Radiation oncologists should cooperate with professional psychologists to reduce FCR by taking online psychological interventions during pandemic.

One of the major limitations of the study is that it was based on patient reported outcome and self-designed single question. There could be a recall bias of the respondents. Also, socio-economic factors, more detailed stages and subtypes of operable BC were ignored for analysis of FCR. Only patients referred for RT are eligible in our study, which could result in sample bias. This probably could explain why demographic variables and systemic treatment strategy do not significantly impact FCR in this study. Moreover, there is no control group prior to the pandemic to be compared in this study. In addition, we haven’t implemented active psychological interventions to alleviate the FCR and improve the QoL of BC patients during the pandemic, thus further studies focusing on this issue are still needed.

In conclusion, our survey confirmed that RT strategy for BC patients has been greatly influenced during the pandemic. Hierarchical multiple regression analysis indicates that RT interruption is an independent predictor for high FCR. The findings of present survey emphasize the necessity to ensure the continuum of RT in BC patients, and efforts should be taken through psychological interventions and on-line professional consultants to alleviate the FCR for BC patients affected by the pandemic. Short course regimens are preferred in clinical practices during pandemic to decrease events of RT interruptions.

## Data Availability Statement

The raw data supporting the conclusions of this article will be made available by the authors, without undue reservation.

## Ethics Statement

The studies involving human participants were reviewed and approved by Ethical Committee of Ruijin Hospital, Shanghai Jiao Tong University, school of medicine. The patients/participants provided their written informed consent to participate in this study.

## Author Contributions

Concept, design, analysis and interpretation of data, manuscript writing: JX, WQ, LC, YT, JH, ZfJ, YuW, XH, JC. Collection of data and final approval of manuscript: JX, WQ, LC, YT, JH, XG, BC, PS, YuZ, YiZ, QZ, HH, YbW, HF, ZjJ, HL, XZ, XQ, FX, DO, SW, CX, ML, ZfJ, YuW, XH, JC. All authors contributed to the article and approved the submitted version,

## Funding

This study was supported in part by the National Natural Science Foundation of China (grant numbers 81673102 to JC, 81602791 to LC, 81803164 to SW, 81972963 to ML); National Key Research and Development Program of China (grant numbers 2016YFC0105409 to JC); Clinical Research Plan of SHDC (grant numbers SHDC2020CR2052B to JC, SHDC2020CR4070 to WQ); Shanghai Municipal Education Commission-Gaofeng Clinical Medicine Grant Support (grant numbers 20171904 to ML); Shanghai Jiaotong University Translational Medicine Fund Support (grant numbers ZH2018QNA54 to DO); Special construction of integrated Chinese and Western medicine in general hospital (grant numbers ZHYY-ZXYJHZ X-2-201704 to CX); and Scientific and Technological Innovation Action Plan of Shanghai Science and Technology Committee (grant numbers 19411950900 and 19411950901 to JC). 

## Conflict of Interest

The authors declare that the research was conducted in the absence of any commercial or financial relationships that could be construed as a potential conflict of interest.

## Publisher’s Note

All claims expressed in this article are solely those of the authors and do not necessarily represent those of their affiliated organizations, or those of the publisher, the editors and the reviewers. Any product that may be evaluated in this article, or claim that may be made by its manufacturer, is not guaranteed or endorsed by the publisher.

## Abbreviations

COVID-19, Novel coronavirus disease; BC, Breast cancer; RT, Radiotherapy; CT, Computed tomography; FCR, Fears of cancer recurrence; CSCO BC, Chinese Society of Clinical Oncology Breast Cancer; FoP-Q-SF, Fear of Progression Questionnaire ⁃Short Form; HADS, Hospital Anxiety and Depression Scale; QoL, quality of life; NAC, neoadjuvant chemotherapy; ANOVA, one-way analysis of variance; SD, standard distance; PST, problem solving therapy.
