# Supplementary material for: Predictors for Fear of Cancer Recurrence in Breast Cancer Patients Referred to Radiation Therapy During the COVID-19 Pandemic: A Multi-Center Cross-Section Survey
Source: Front Oncol. 2021 Jul 26;11:650766. doi: 10.3389/fonc.2021.650766 (PMC8351463; doi:10.3389/fonc.2021.650766)
Supplement: Supplementary file 1 [file DataSheet_1.pdf]

新冠病毒肺炎疫情所致非预期中断或延迟放疗对乳腺癌患者心理健康影响的评估

表 1：基本情况

姓名 年龄 性别

是否在职 受教育程度 确诊时间:

婚姻状态 初诊医院:

手术医院（若无手术，填无）:

放疗医院（若未开始放疗，请填写就诊的放疗门诊医院或无）:

填写日期:

您目前的肿瘤情况：

1. 初治乳腺肿瘤（12-15 题无需填写）:

2. 复发转移肿瘤（3-11 题无需填写）: （1）局部区域复发（指乳房或胸壁以及淋巴结复发）；（2）远处转移；（3）局部区域复发和远处转移

若为初治肿瘤，

3.疫情发生时（2020 农历新年）您已完成的治疗方法（多选）: 手术 化疗 放疗 靶向治疗 内分泌治疗

4.疫情发生时您正在接受的抗肿瘤治疗（多选）: 手术 化疗 放疗 靶向治疗 内分泌治疗

5.若您已完成手术，您的手术方式: 1.乳房切除；2.保乳术；

您的手术时间: ____________________

6.术后化疗情况: 1. 已完成；2.未完成；3.计划化疗未开始化疗 4.无需化疗

末次化疗时间（未化疗无需填写）: ____________

7.目前您正在接受靶向药物: 1.有；2.无；计划接受靶向治疗: 1.有；2.无；

8.目前您正在内分泌治疗: 1.有；2.无；计划内分泌治疗: 1.是；否

9.疫情开始时（2020 农历新年），您处于放射治疗的哪一阶段: 未开始

已开始　　　结束

10.您在疫情期间放射治疗情况：治疗尚未进入放疗阶段 按原放疗计划治疗　　中断　　　　其他 （请描述）_____

若您疫情开始期间放射治疗受到中断，计划放疗___次，中断时已完成___次，中断._____天后恢复

11.目前您处于放射治疗的哪一阶段：未开始　　已开始　　　结束

若为复发转移肿瘤，

12.疫情发生时您正在接受的抗肿瘤治疗（多选）：手术　放疗 化疗　靶向治疗　内分泌治疗　免疫治疗；

13.您接受放射治疗的部位：（可多选）

乳房/胸壁　　颈部和/或者腋窝淋巴结　　脑转移　骨转移　其他部位

14.疫情开始期间（2020 农历新年）您处于放射治疗的哪一阶段：未开始　已开始　　　结束

15.您在疫情期间放射治疗情况：按原方案治疗　　　　中断　　　　其他

若您疫情开始期间放射治疗受到中断，计划放疗___次，中断时已完成___次，中断._____天后恢复

目前您处于放射治疗的哪一阶段：未开始　　已开始　　　结束

治疗中断疫情相关因素

16.若您在疫情期间放射治疗计划受到影响，请问发生的具体时间是在何时：（以**月**旬填写，例如 2 月上旬）

17.您觉得疫情期间放射治疗中断或延迟原因来源于哪一方面(可多选)：

1) 交通运输管控；
2) 就诊医院或科室限制肿瘤人员诊治；
3) 个人原因，担心在医院被感染；
4) 不愿意接受医院的新冠肺炎筛查（例如每次住院需要复查胸部 CT 等）；
5) 医院床位收治数目因新冠疫情而受到限制；
6) 其他（请描述）

18.本人是否确诊感染新冠肺炎：是　　否

19.家属是否有确诊新冠肺炎： 是　　　否

20.您所在居住地现在对外交通是否恢复正常：是　　否　　不清楚

21.您手术的医院现在对外地患者是否有限制：是　　否　　不清楚

22.您放疗的医院现在对外地患者是否有限制：是　　否　　不清楚

癌症患者恐惧疾病进展量表 (FoP-Q-SF)

填表注意事项:下面有二十条文字（括号中为症状名称），请仔细阅读每一条,

把意思弄明白，每一条文字后有五级评分,然后根据您最近一星期的实际情况，在分数栏 1～5 分适当的分数下划“√”。

	1 没有	2 偶尔	3 有时	4 经常	5 总是如此
1．想到疾病可能会进展，我变得焦虑					
2. 在医生预约或定期检查前我感到紧张					
3．我害怕此病引起的疼痛					
4．因疾病降低工作效率的想法使我烦恼					
5．当我焦虑时会有一些身体不适，如心跳加快、胃痛、紧张等					
6．我担心我的病可能会传给我孩子					
7．我的日常生活可能不得不依靠陌生人，这使我焦虑					
8．我担心某些时候因病不能再继续自己得爱好/嗜好					
9．我担心疾病过程中会有一些重大得治疗					
10．我担心药物会损害我的身体					
11．我担心如果我发生什么事情，家庭会怎么样					
12．因病可能无法工作得想法使我烦恼					

HADS 量表（医院用焦虑抑郁量表）

情绪在大多数疾病中起着重要作用，如有医生了解您的情绪变化，他们就能给您更多的帮助，请您阅读以下各个项目，在其中最符合你过去一个月的情绪情况选项后括号内打”√”。对这些问题的回答不要做过多的考虑，立即做出的回答往往更符合实际情况。

1.我感到紧张（或痛苦）（A）

几乎所有时候 3

大多数时候 2

有时 1

根本没有 0

2.我对以往感兴趣的事情还是有兴趣(D)

肯定一样 0

不像以前那样多 1

只有一点儿 2

基本上没有了 3

3.我感到有点害怕，好像预感到有什么可怕事情要发生（A）

非常肯定和十分严重 3

是有，但并不太严重 2

有一点，但并不使我苦恼 1

根本没有 0

4.我能够哈哈大笑，并看到事物好的一面(D)

我经常这样 0

现在已经不大这样了 1

现在肯定是不太多了 2

根本没有 3

5.我的心中充满烦恼（A）

大多数时间 3

常常如此 2

时时，但并不经常 1

偶然如此 0

6.我感到愉快（D）

根本没有 3

并不经常 2

有时 1

大多数 0

7.我能够安闲而轻松地坐着（A）

肯定 0

经常 1

并不经常 2

根本没有 3

8.我对自己的仪容（打扮自己）失去兴趣（D）

肯定 3

并不像我应该做到的那样关心 2

我可能不是非常关心 1

我仍像以往一样关心 0

9.我有点坐立不安，好像感到非要活动不可(A)

确实非常多 3

是不少 2

并不很多 1

根本没有 0

10. 我对一切都是乐观地向前看（D）

差不多是这样做的 0

并不完全是这样做的 1

很少这样做 2

几乎从来不这样做 3

11.我突然发现恐慌感(A)

确实很经常 3

时常 2

并非经常 1

根本没有 0

12.我好像感到情绪在渐渐低落(D)

几乎所有的时间 3

很经常 2

有时 1

根本没有 0

13.我感到有点害怕，好像某个内脏器官变坏了（A）

根本没有 0

有时 1

很经常 2

非常经常 3

14.我能欣赏一本好书或一项好的广播或电视节目(D)

常常 0

有时 1

并非经常 2

很少 3

EORTC QLQ-C30 (version 3) 生活质量调查问卷

我们很希望了解一些有关您及您的健康状况的信息。请独立回答以下所有问题，并圈出对您最合适的答案。答案无“正确”、与“错误”之分。您提供的信息我们将绝对保密。

	没有	有一点	有一些	非常多
1.当您做一些费力的动作，如提沉重的购物袋或行李箱时，您是否感到困难？	1	2	3	4
2.长距离步行时，您是否感到困难？	1	2	3	4
3.在户外短距离散步时，您是否感到困难？	1	2	3	4
4.在白天，您是否必须卧床或坐在椅子上？	1	2	3	4
5.您是否需要别人协助进食、穿衣、洗漱或上厕所？	1	2	3	4

**在过去的一周中：**	**没有**	**有一点**	**有一些**	**非常多**
6.您的工作或者日常活动是否受到体能限制？	1	2	3	4
7.您的业余爱好和休闲活动是否受到体能限制？	1	2	3	4
8.您曾感到气短吗？	1	2	3	4
9.您有过疼痛吗？	1	2	3	4
10.您曾需要休息吗？	1	2	3	4
11.您曾感到睡眠不好吗？	1	2	3	4
12.您曾感到虚弱吗？	1	2	3	4
13.您曾感到没有胃口吗？	1	2	3	4

14.您曾感受到恶心想吐吗？	1	2	3	4
15.您曾呕吐过吗？	1	2	3	4
16.您曾有便秘吗？	1	2	3	4

**在过去的一周中：**	**没有**	**有一点**	**有一些**	**非常多**
17.您曾有过腹泻？	1	2	3	4
18.您曾感觉疲乏吗？	1	2	3	4
19.疼痛妨碍您的日常活动吗？	1	2	3	4
20.您是否很难集中注意力做事，例如读报或看电视？	1	2	3	4
21.您曾感到紧张吗？	1	2	3	4
22.您曾感到担心吗？	1	2	3	4
23.您曾感到容易动怒吗？	1	2	3	4
24.您曾感到情绪低落吗？	1	2	3	4
25.您曾经感到记事困难吗？	1	2	3	4
26.您的身体状况或治疗过程，妨碍了您的家庭生活吗？	1	2	3	4
27.您的身体状况或治疗过程，妨碍了您的社交活动吗？	1	2	3	4
28.您的身体状况或治疗过程，造成了您的经济困难吗？	1	2	3	4

以下问题，数字 1-7 代表从“很差”到“很好”的等级，请在 1 至 7 之间圈出对您最合适的答案。

29.您如何评定过去一周中您的整体健康状况？

1	2	3	4	5	6	7
很差						很好

30.您如何评定过去一周中您的整体生活质量？

1	2	3	4	5	6	7
很差						很好

English version for questionnaire in part

The survey for the psychological influence on breast cancer patients referred to radiation therapy during COVID-19 pandemic

Figure 1：basic characteristic

Name: Age: Sex :

Employed or not: education level:

diagnosis date of breast cancer:

married or not: hospital for diagnosis:

hospital for surgery（if not，write ‘No’）：

hospital for radiation therapy（if have not start the radiation therapy, write ‘No’ or the planned hospital for radiation therapy）：

date：

Stage of tumor：

1. operable（Q12-15 is not needed to answer）：

2. recurrence or metastatic （Q3-11 is not needed to answer： （1）local （recurrence of ipsilateral breast or chest wall or lymph nodes）； （2）distant； （3）local and distant

If ‘stage of tumor’ is ‘operable’,

3.what type of anti-cancer therapy had been finished before the Spring Festival of 2020 (multiple choice)? (1) chemotherapy (2) radiation therapy (3) target therapy (4) endocrine therapy

4.which type of anti-cancer therapy you received during the pandemic：手 (1) chemotherapy (2) radiation therapy (3) target therapy (4) endocrine therapy (5) surgery

5.what is type of surgery and the date of surgery：(1) mastectomy；(2)

breast conserving therapy. date of your surgery: ————————————

6. Do you receive chemotherapy: (1) finished; (2) not finished (3) plan to receive but have not started (4) not needed

Date of last cycle of chemotherapy: ——————

7.Do you receive target therapy now: (1) yes (2) not needed (3) plan to receive but have not started

8. Do you receive endocrine therapy now: (1) yes (2) not needed (3) plan to receive but have not started

9. What stage of RT procedure were you on during the Spring Festival of 2020 (peak of pandemic in China) and today, respectively? (1) planned to RT (2) undergoing (3) completed;

10. Was your RT schedule modified during the pandemic? (1) continued as planned (2) interrupted (3) others;

How many days was your RT interrupted?

11. Do you receive target therapy now: (1) Yes (2)No but finished (2)No but have not started

If 'stage of tumor' is 'recurrence or metastatic',

12. what type of anti-cancer therapy had been finished before the Spring Festival of 2020 (multiple choice)? (1) chemotherapy (2) radiation therapy (3) target therapy (4) endocrine therapy

13.Regions of radiation therapy : (multiple choice)

(1) breast/ chest wall (2) axillary lymph nodes or cervical lymph node (3) brain (4) bone (5) others

14. What stage of RT procedure were you on during the Spring Festival of 2020 (peak of pandemic in China) and today, respectively? (1) planned to RT (2) undergoing (3) completed;

15. Was your RT schedule modified during the pandemic? (1) continued as planned (2) interrupted (3) others;

How many days was your RT interrupted?

Do you receive target therapy now: (1) Yes (2)No but finished (2)No but

have not started

Reasons for delay or interruptions of radiation therapy

16. If your RT schedule was impacted by COVID-19, could you tell the detailed time?

17. Did you think that your RT schedule had been impacted by pandemic? Which of the following reasons account for the interruption or delayed RT: (1) aggressive quarantine measures (2) restricted number of cancer patients admitted for hospital under the constraints of social distancing and local guidelines (3) personal reason, afraid of being infected by COVID-19 (4) declined to receive repeated COVID-19 screenings including chest CT scan (5) restriction of number of beds in hospital due to COVID-19 (6) others;

18. Whether you were diagnosed as COVID-19? (1) Yes (2) No

19. Whether your family members were diagnosed as COVID-19? (1) Yes (2) No

20. Whether your city had the restriction for traffic? (1) Yes (2) No (3)not known

21. Whether the surgery hospital had the restrictions for patients from other cities? (1) Yes (2) No (3) not known

22. Whether the radiation therapy hospital had the restrictions for patients from other cities? (1) Yes (2) No (3) not known

Questionnaires:

Fear of Progression Questionnaire - Short Form

Hospital Anxiety and Depression Scale

EORTC QLQ-C30
