# Supplementary material for: Predictors for Fear of Cancer Recurrence in Breast Cancer Patients Referred to Radiation Therapy During the COVID-19 Pandemic: A Multi-Center Cross-Section Survey
Source: Front Oncol. 2021 Jul 26;11:650766. doi: 10.3389/fonc.2021.650766 (PMC8351463; doi:10.3389/fonc.2021.650766)
Supplement: Supplementary file 2 [file DataSheet_2.doc]

**Additional File 2**

**Columns**

Page 2: Table S1. Reasons of influence on RT during COVID-19 pandemic in affected and interruptions patients

Page 3: Table S2: Comparisons with the time to radiotherapy during the same time in 2019

Page 4-5: Table S3. Detailed univariate analysis on associated factors with FCR scores

Page 5: Table S4. Correlation (Pearson’s r) with the FoP-Q-SF for the validation scales.

Page 6-7: Table S5. The hierarchical multiple regression model of FCR

Page 8: Fig S1. Flow chart and distribution of influence on RT strategy

Page 9: Fig S2. Mean score of each item in FoP-Q-SF among different influences on radiotherapy schedule

Page 9: Fig S3. Comparisons of FCR in hospital volumes, regions and influence on RT schedule;

**Table S1. Reasons of influence on RT during COVID-19 pandemic in affected and interruptions patients**

| **Reasons** | **Total (*N*=265)**  ***N* (%)** | **Interruptions (*N*=24)**  ***N* (%)** | **Guangdong Province（*N*=147）**  ***N* (%)** | **Shanxi Province（*N*=76）**  ***N* (%)** | **Yangtze River delta region（*N*=42）**  ***N* (%)** |
| --- | --- | --- | --- | --- | --- |
| Aggressive quarantine measures | 65(24.5) | 4(16.7) | 14(9.5) | 30(39.5) | 21(50) |
| Restricted number of cancer patients admitted for hospital under the constraints of social distancing and local guidelines | 165(62.3) | 18(75.0) | 123(83.7) | 34(44.7) | 7(16.7) |
| Personal reason, afraid of being infected by the COVID-19 | 78(29.4) | 13(54.2) | 49(33.3) | 15(19.7) | 15(35.7) |
| declined to receive repeated COVID-19 screenings including chest CT scan | 13(4.9) | 3(12.5) | 10(6.8) | 1(1.3) | 5(11.9) |
| Restriction of number of beds in hospital due to COVID-19 | 92(34.7) | 7(29.2) | 34(23.1) | 54(71) | 4(9.5) |
| Others ^a^ | 19(7.2) | 0(0) | 6(4.1) | 3(3.9) | 10(23.8) |

a: being refused by hospital due to fever, cannot remove the subcutaneous port and so on.

Abbreviations: RT=radiotherapy, CT= computed tomography

**Table S2: Comparisons with the time to radiotherapy during the same period in 2019**

| **Time to RT** | **The Yangtze River Delta Region** | | **Guangdong province** | | **Shanxi province** | |
| --- | --- | --- | --- | --- | --- | --- |
|  | **2019** | **2020** | **2019** | **2020** | **2019** | **2020** |
| **Patients, *N*** | 165 | 60 | 80 | 59 | 51 | 34 |
| **Mean(days)** | 21.2 | 20.2 | 42.7 | 74.7 | 39.7 | 61.9 |
| **SD (days)** | 10.4 | 12.8 | 12.4 | 23.2 | 14.7 | 42.3 |
| ***p*** | 0.104 | | ＜0.001 | | 0.006 | |
| **≤4 weeks**  ***N (%)*** | 136(82.4) | 51(85) | 10(12.5) | 4(6.8) | 9(17.6) | 8(23.5) |
| **4-8 weeks**  ***N (%)*** | 27(16.4) | 9(15) | 61(76.3) | 8(13.6) | 40(78.4) | 10(29.4) |
| **8-12 weeks**  ***N (%)*** | 1(0.6) | 0(0) | 9(11.3) | 19(32.2) | 0(0) | 5(14.7) |
| **≥12 weeks**  ***N (%)*** | 1(0.6) | 0(0) | 0(0) | 28(47.5) | 2(3.9) | 11(32.4) |
| ***p*** | 0.914 | | ＜0.001 | | ＜0.001 | |

* Abbreviations: RT = radiotherapy from last dose of chemotherapy

**Table S3: Univariate analysis on associa**ted factors with FCR scores

| **Factors** | | **Whole cohort (*N*=488)** | | | | **Patients with High-level FCR (*N*=84)** | |
| --- | --- | --- | --- | --- | --- | --- | --- |
|  |  | ***N*** | **Mean** | **SD** | ***p*** | ***N*** | **%^a^** |
| Age, y | |  |  |  | 0.44 |  |  |
|  | ≤40 | 88 | 25.76 | 8.064 |  | 16 | 18.2 |
|  | 41~60 | 313 | 24.49 | 8.669 |  | 53 | 16.9 |
|  | ＞60 | 87 | 25.13 | 8.636 |  | 15 | 17.2 |
| Sex | |  |  |  | 0.978 |  |  |
|  | Female | 486 | 24.83 | 8.571 |  | 84 | 17.3 |
|  | Male | 2 | 25 | 2.828 |  | 0 | 0 |
| Employment status | |  |  |  | 0.483 |  |  |
|  | Employed | 168 | 25.19 | 7.703 |  | 58 | 18.1 |
|  | unemployed | 320 | 24.64 | 8.975 |  | 26 | 15.5 |
| Education | |  |  |  | 0.667 |  |  |
|  | High school and below | 341 | 25.03 | 8.862 |  | 65 | 19.1 |
|  | bachelor | 138 | 24.46 | 7.934 |  | 19 | 13.8 |
|  | Master and above | 9 | 23.11 | 5.645 |  | 0 | 0 |
| Marriage | |  |  |  | 0.382 |  |  |
|  | Single | 13 | 21.69 | 4.59 |  | 0 | 0 |
|  | Married | 474 | 24.91 | 8.632 |  | 84 | 17.1 |
|  | widowed | 1 | 28 | 0 |  | 0 | 0 |
| Hospital volume | |  |  |  | 0.006* |  |  |
|  | ＜100 BC cases | 54 | 29.04 | 10.233 |  | 16 | 29.6 |
|  | 100~499 BC cases | 165 | 24.52 | 7.829 |  | 27 | 16.4 |
|  | ≥500 cases BC | 269 | 24.18 | 8.409 |  | 41 | 15.2 |
| Region | |  |  |  | 0.038* |  |  |
|  | The Yangtze River Delta Region | 191 | 24.34 | 7.676 |  | 29 | 15.2 |
|  | Guangdong province | 193 | 26 | 8.742 |  | 39 | 20.2 |
|  | Shanxi province | 104 | 23.57 | 9.5 |  | 16 | 15.4 |
| Stage of tumor | |  |  |  | 0.154 |  |  |
|  | operable BC | 446 | 24.62 | 8.328 |  | 68 | 15.2 |
|  | recurrent or metastatic BC | 42 | 27.05 | 10.532 |  | 16 | 38.1 |
| Chemotherapy | |  |  |  | 0.865 |  |  |
|  | Yes | 414 | 24.86 | 8.671 |  | 70 | 16.9 |
|  | No | 74 | 24.68 | 7.924 |  | 14 | 18.9 |
| Target therapy | |  |  |  | 0.205 |  |  |
|  | Yes | 128 | 24.01 | 8.074 |  | 20 | 15.6 |
|  | No | 360 | 25.13 | 8.711 |  | 64 | 17.8 |
| Endocrine therapy | |  |  |  | 0.103 |  |  |
|  | Yes | 329 | 25.27 | 8.856 |  | 60 | 18.2 |
|  | No | 159 | 23.92 | 7.842 |  | 24 | 15.1 |
| RT procedure | |  |  |  | 0.985 |  |  |
|  | Completed | 143 | 24.94 | 8.137 |  | 23 | 16.1 |
|  | Undergoing | 268 | 24.78 | 8.471 |  | 47 | 17.5 |
|  | Planned to RT | 77 | 24.83 | 9.646 |  | 14 | 18.2 |
| Influence of RT schedule | |  |  |  | ＜0.001* |  |  |
|  | normal | 242 | 23.96 | 7.854 |  | 32 | 13.2 |
|  | Delay of RT | 149 | 23.81 | 7.853 |  | 17 | 11.4 |
|  | Interruption of RT | 24 | 30.75 | 8.759 |  | 12 | 50 |
|  | Special normal | 73 | 27.88 | 10.555 |  | 23 | 31.5 |
| Change of hospital level | |  |  |  | 0.009* |  |  |
|  | Down | 32 | 28.63 | 9.387 |  | 8 | 25 |
|  | Up or no change | 456 | 24.57 | 8.44 |  | 76 | 16.7 |

*:p＜0.05

a: the percent = number of patients with high-level FCR/ total number of patients in each subgroup

Abbreviations: FCR=fear of cancer recurrence，BC=breast cancer, BCS=breast conserving surgery, RT=radiotherapy

**Table S4. Correlation (Pearson’s r) with the FoP-Q-SF for the validation scales.**

| **Scale** | | **M** | **SD** | **r** | ***P**** |
| --- | --- | --- | --- | --- | --- |
| QLQ-C30 | |  |  |  |  |
|  | Physical function | 85.02 | 13.31 | -0.349 | ＜0.001 |
|  | Role function | 82.81 | 16.92 | -0.346 | ＜0.001 |
|  | Emotional function | 77.91 | 15.85 | -0.598 | ＜0.001 |
|  | Cognitive function | 82.4 | 15.78 | -0.43 | ＜0.001 |
|  | Social function | 78.64 | 18.27 | -0.506 | ＜0.001 |
|  | Global quality of life | 61.79 | 16.50 | -0.341 | ＜0.001 |
| HADS | |  |  |  |  |
|  | Anxiety | 4.97 | 3.26 | 0.701 | ＜0.001 |
|  | Depression | 5.3 | 4.16 | 0.577 | ＜0.001 |

* *p* was calculated by Pearson correlation analyses.

**Table S5: T**he hierarchical multiple regression model of FCR

| **Variables** | | **Model 1** | | | **Model 2** | | | **Model 3** | | | **Model 4** | | |
| --- | --- | --- | --- | --- | --- | --- | --- | --- | --- | --- | --- | --- | --- |
|  |  | **Unadjusted B** | **Adjusted β** | ***p*** | **Unadjusted B** | **Adjusted β** | ***p*** | **Unadjusted B** | **Adjusted β** | ***p*** | **Unadjusted B** | **Adjusted β** | ***p*** |
| Regions | |  |  |  |  |  |  |  |  |  |  |  |  |
|  | Guangdong province | (Reference) | (Reference) |  | (Reference) | (Reference) |  | (Reference) | (Reference) |  | (Reference) | (Reference) |  |
|  | The Yangtze River delta region | -0.377 | -0.022 | 0.69 | -0.913 | -0.052 | 0.215 | -0.466 | -0.027 | 0.469 | -0.133 | -0.008 | 0.859 |
|  | Shanxi province | -1.862 | -0.089 | 0.078 | -2.691 | -0.129 | 0.001* | -2.323 | -0.111 | 0.001* | -2.432 | -0.117 | 0.001* |
| Hospital volume | |  |  |  |  |  |  |  |  |  |  |  |  |
|  | ≥500 BC cases | (Reference) | (Reference) |  | (Reference) | (Reference) |  | (Reference) | (Reference) |  | (Reference) | (Reference) |  |
|  | 100~499 BC cases | 0.038 | 0.002 | 0.965 | 0.22 | 0.012 | 0.745 | -0.961 | -0.053 | 0.111 | -1.16 | -0.064 | 0.073 |
|  | ＜100 BC cases | 4.525 | 0.166 | 0.001* | 2.239 | 0.082 | 0.03* | 1.676 | 0.062 | 0.067 | 1.514 | 0.056 | 0.122 |
| Stage of tumor | | 2.908 | 0.095 | 0.039* | 1.588 | 0.052 | 0.147 | 1.142 | 0.037 | 0.233 | 0.745 | 0.024 | 0.444 |
| Endocrine therapy | | 1.321 | 0.072 | 0.115 | 1.191 | 0.065 | 0.063 | 0.894 | 0.049 | 0.111 | 0.814 | 0.045 | 0.145 |
| QLQ-C30 | |  |  |  |  |  |  |  |  |  |  |  |  |
|  | Physical function | N/A | N/A | N/A | -0.025 | -0.038 | 0.411 | -0.009 | -0.014 | 0.726 | -0.005 | -0.007 | 0.859 |
|  | Role function | N/A | N/A | N/A | 0.012 | 0.024 | 0.604 | 0.002 | 0.005 | 0.909 | 0.006 | 0.012 | 0.762 |
|  | Emotional function | N/A | N/A | N/A | -0.222 | -0.411 | ＜0.001* | -0.107 | -0.198 | ＜0.001* | -0.103 | -0.19 | ＜0.001* |
|  | Cognitive function | N/A | N/A | N/A | -0.045 | -0.082 | 0.058 | -0.022 | -0.041 | 0.281 | -0.025 | -0.046 | 0.221 |
|  | Social function | N/A | N/A | N/A | -0.091 | -0.195 | ＜0.001* | -0.054 | -0.116 | 0.005* | -0.052 | -0.111 | 0.006* |
|  | Global quality of life | N/A | N/A | N/A | -0.054 | -0.105 | 0.008* | -0.025 | -0.048 | 0.172 | -0.029 | -0.056 | 0.106 |
| HADS | |  |  |  |  |  |  |  |  |  |  |  |  |
|  | Anxiety | N/A | N/A | N/A | N/A | N/A | N/A | 1.163 | 0.443 | ＜0.001* | 1.138 | 0.434 | ＜0.001* |
|  | Depression | N/A | N/A | N/A | N/A | N/A | N/A | 0.118 | 0.057 | 0.227 | 0.113 | 0.055 | 0.244 |
| Influence on RT schedule | |  |  |  |  |  |  |  |  |  |  |  |  |
|  | Normal | N/A | N/A | N/A | N/A | N/A | N/A | N/A | N/A | N/A | (Reference) | (Reference) |  |
|  | Delay | N/A | N/A | N/A | N/A | N/A | N/A | N/A | N/A | N/A | -0.179 | -0.01 | 0.808 |
|  | Interruption | N/A | N/A | N/A | N/A | N/A | N/A | N/A | N/A | N/A | 2.787 | 0.071 | 0.035* |
|  | Special normal | N/A | N/A | N/A | N/A | N/A | N/A | N/A | N/A | N/A | 1.546 | 0.065 | 0.06 |
| Change of hospital level | | N/A | N/A | N/A | N/A | N/A | N/A | N/A | N/A | N/A | 1.83 | 0.053 | 0.09 |
| R^2^ | | 0.05 | | | 0.456 | | | 0.587 | | | 0.597 | | |
| Adjust R^2^ | | 0.038 | | | 0.442 | | | 0.574 | | | 0.581 | | |
| F | | 4.182 | | | 33.133 | | | 47.959 | | | 38.581 | | |
| Sig. of model | | ＜0.001* | | | ＜0.001* | | | ＜0.001* | | | ＜0.001* | | |
| △R^2^ | | 0.05 | | | 0.406 | | | 0.131 | | | 0.01 | | |
| △F | | 4.182 | | | 59.055 | | | 74.987 | | | 2.966 | | |
| Sig. of △F | | ＜0.001* | | | ＜0.001* | | | ＜0.001* | | | 0.019* | | |

*: P<0.05; Abbreviations: N/A=not applicable, FCR=fear of cancer recurrence，BC=breast cancer, RT=radiotherapy, Sig.=significance

Coding information：Stage of tumor: recurrent or metastatic BC=1, operable BC=0; Endocrine therapy: Yes=1, No=0

**Fig S1. Flow chart and distribution of influence on RT strategy**


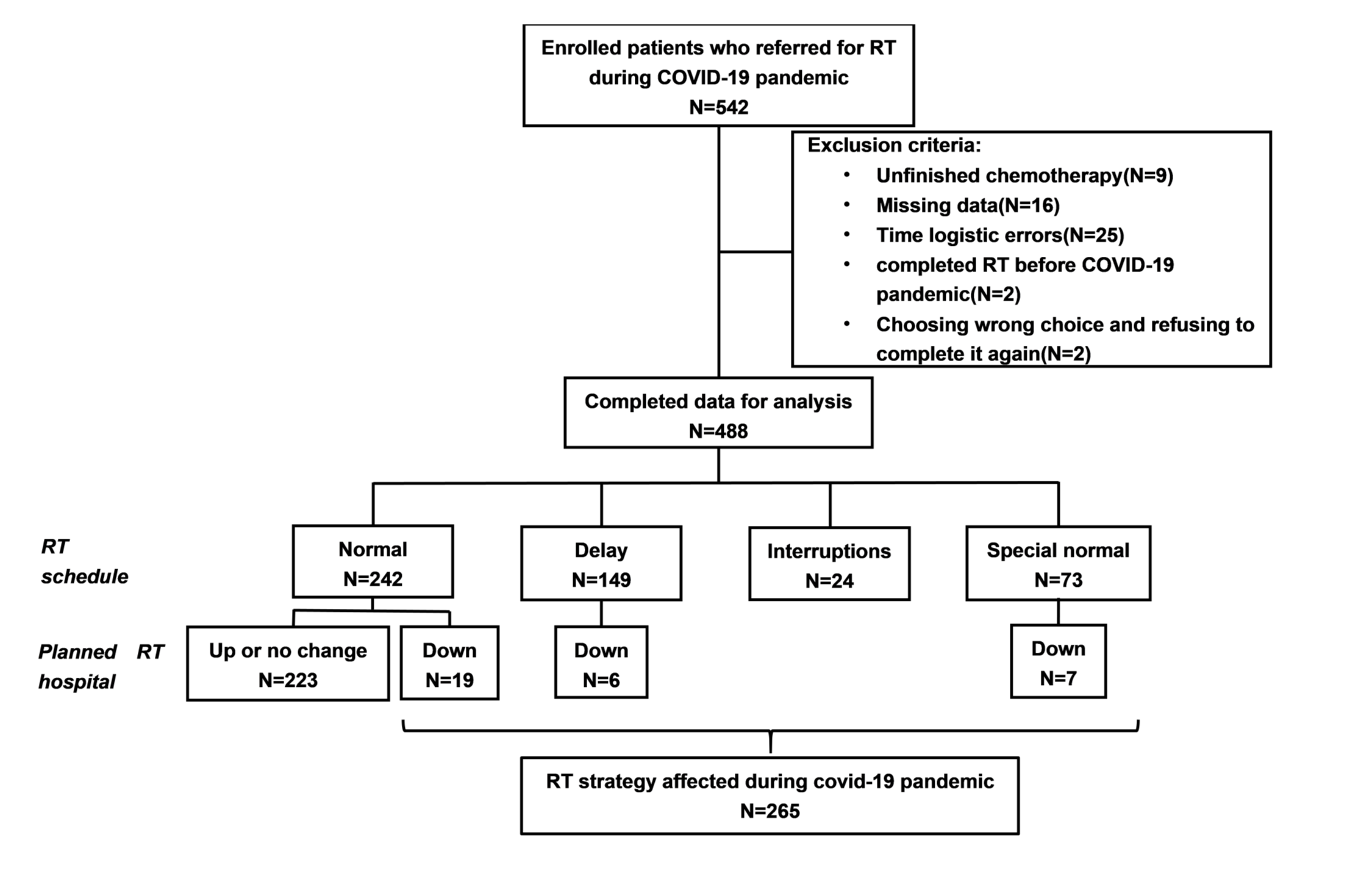


Abbreviations: RT=radiotherapy

**Fig S2 Mean score of each item in FoP-Q-SF among different influences on radiotherapy schedule**

**
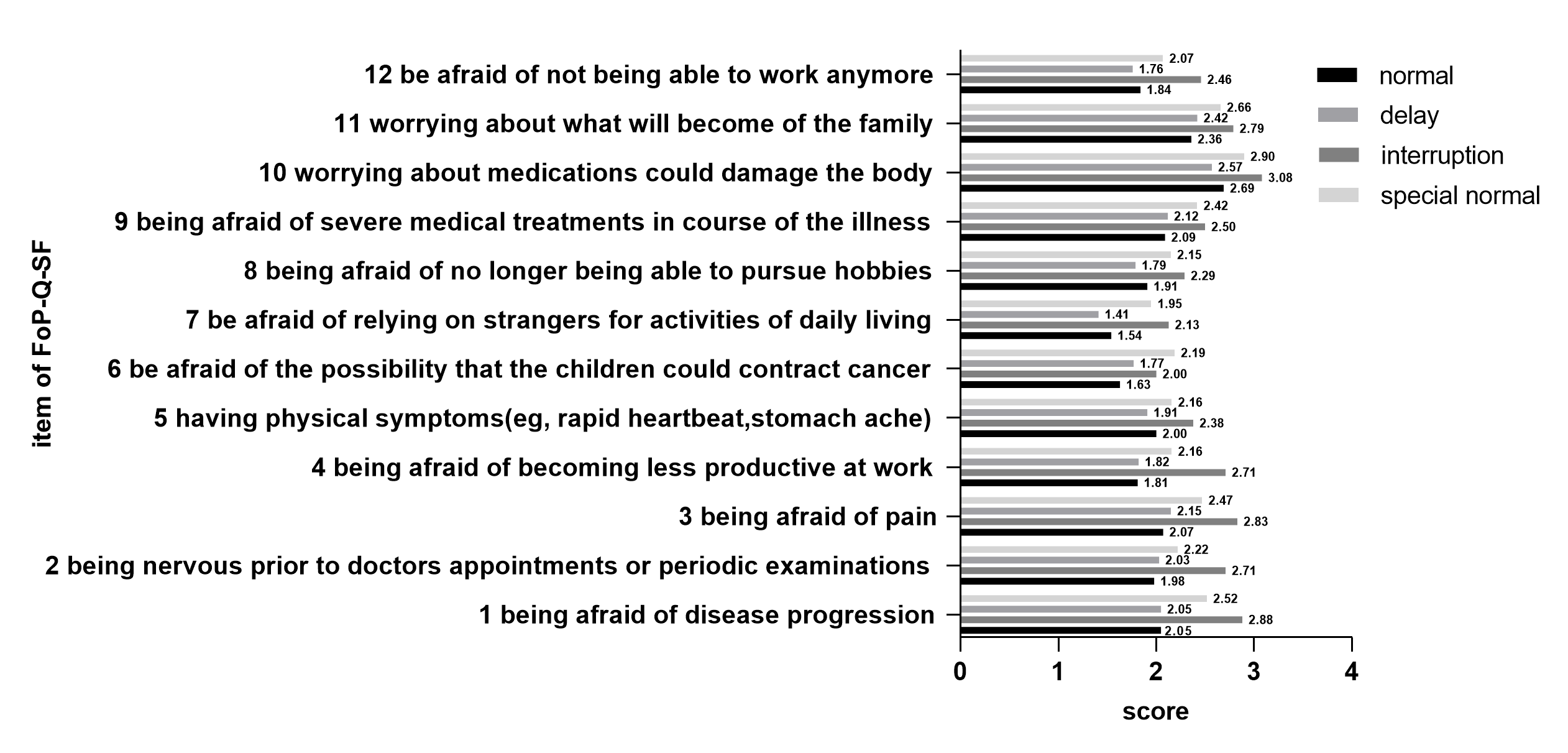
**

**Fig S3 Comparisons of FCR in hospital volumes, regions and influence on RT schedule**

**
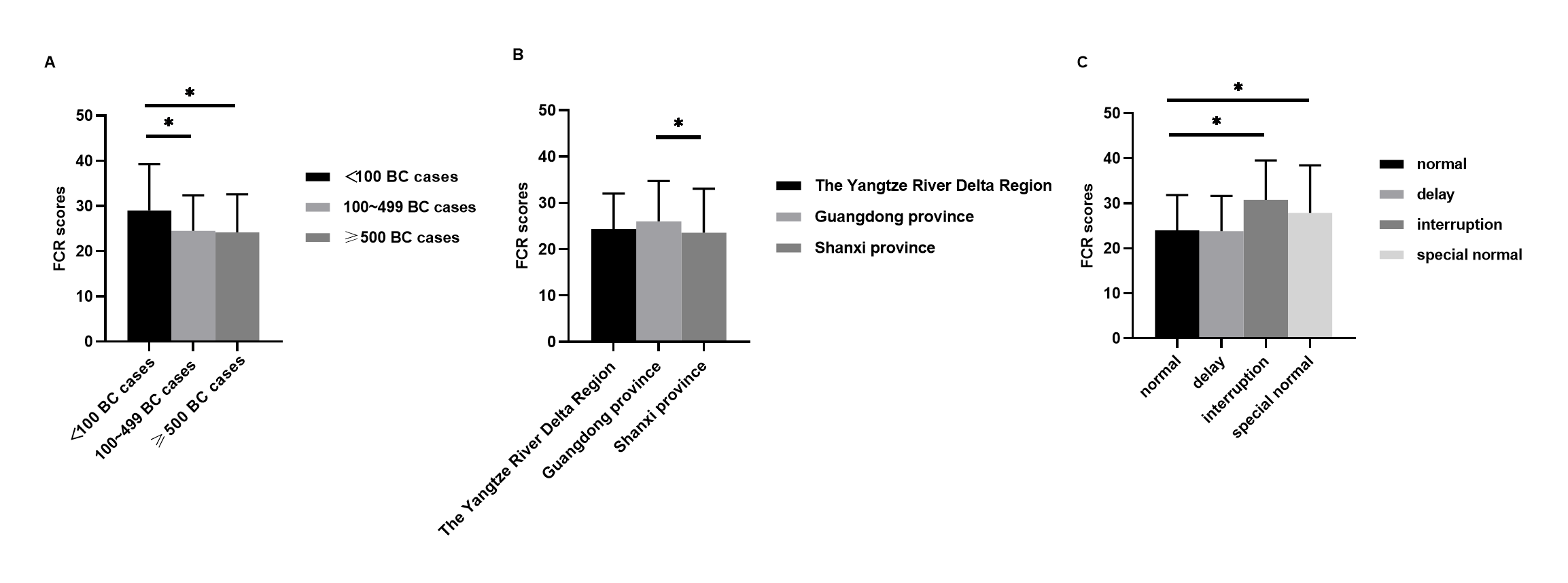
**

(A) hospital volumes, (B) regions, (C) influence on RT schedule

* p＜0.05

Abbreviations: BC=breast cancer, FCR=fear of cancer progression;
